# Pain in IBD Patients: Very Frequent and Frequently Insufficiently Taken into Account

**DOI:** 10.1371/journal.pone.0156666

**Published:** 2016-06-22

**Authors:** Jonas Zeitz, Melike Ak, Séverine Müller-Mottet, Sylvie Scharl, Luc Biedermann, Nicolas Fournier, Pascal Frei, Valerie Pittet, Michael Scharl, Michael Fried, Gerhard Rogler, Stephan Vavricka, Swiss IBD Cohort Study Group

**Affiliations:** 1 Division of Gastroenterology and Hepatology, University Hospital Zurich, University of Zurich, Zurich, Switzerland; 2 Division of Pulmonology, Zurcher Rehabilitation Center Wald, Wald, Switzerland; 3 Institute of Social and Preventive Medicine, Université de Lausanne, Lausanne, Switzerland; 4 Gastroenterology Bethanien, Zurich, Switzerland; 5 Zurich Center for Integrative Human Physiology, University of Zurich, Zurich, Switzerland; 6 Division of Gastroenterology, Triemli Spital, Zurich, Switzerland; University Hospital Llandough, UNITED KINGDOM

## Abstract

**Background:**

Pain is a common symptom related to inflammatory bowel disease (IBD). In addition to abdominal pain, pain can also be an extraintestinal manifestation of IBD. Pain treatment is challenging and a substantial part of IBD patients are treated with opioids. Therefore, a better knowledge on pain symptoms is crucial for a better therapeutic approach to this clinical problem.

**Methods:**

Patients of the Swiss IBD Cohort Study (SIBDCS) (n = 2152) received a questionnaire regarding pain intensity, pain localization and impact of pain on daily life and social activities. Furthermore, the questionnaire investigated the use of pain-specific medication.

**Results:**

A vast majority of patients (71%) experienced pain during the disease course. For a substantial part of patients (49% in UC and 55% in CD) pain is a longstanding problem (>5 years). Pain in UC was of shorter duration compared to CD (p < 0.01). Abdominal pain (59.5%) and back pain (38.3%) were the main pain localizations. 67% of patients took pain medication; 24% received no pain treatment. The general quality of life was significantly lower in patients suffering of pain compared to those without pain (38 vs. 77; (-100 very bad; 100 very good) p<0.0001).

**Conclusions:**

Prevalence of pain is high in patients of the SIBDCS. It is a longstanding problem for the majority of the patients affected. Pain was found to be undertreated in the SIBDCS and was significantly associated with health-related quality of life. Thus, an increased awareness is mandatory to address this frequent complication in the course of IBD.

## Introduction

Abdominal pain is a common symptom related to Crohn’s disease (CD) and ulcerative colitis (UC), also collectively known as inflammatory bowel disease (IBD). During disease flares pain is present in 50–70% of IBD patients[1, 2]. Pain can also be caused by extraintestinal manifestations (EIM) of IBD or can be an extraintestinal manifestation by itself and more than 40% of IBD patients suffer from EIM[3–5] With a prevalence in the literature of 1–46%, arthropathies are the most common EIM of IBD[4, 6–16].

Pronounced severe impact of pain on health related quality of life (HRQOL) is known for various diseases[17–20]. Longstanding pain leads to a significant decrease in HRQOL, increase in pain medication intake and comorbidities including depression, anxiety and addiction [21–23]. Lower HRQOL also has been shown to result in an increased healthcare utilization among youth with IBD[24]. Furthermore, pain attacks severely interfere with social and working habits [25–27]. In an analysis of the data from the SIBDCS it could be shown that EIM were one of the most important predictors for temporary work disability in patients with CD[28].

In a German study including some 300 patients using a specifically designed questionnaire regarding pain and HRQOL 87.9% of IBD patients reported pain and showed a significantly reduced HRQOL. Surgery reduced pain and patients on analgesics reported more pain and lower HRQOL than patients not on analgesics [29].

Pain may directly be caused by inflammation as inflammatory cytokines and mediators have been shown to sensitize primary afferent neurons[30]. But ongoing inflammation does not fully explain pain in many IBD patients: about 20% of patients in complete clinical and endoscopic remission continue to experience pain. Pain treatment is complex and challenging. Pain perception in IBD patients may be influenced by multiple factors; peripheral, central, and environmental (stress) factors [31]. Pain treatment is challenging. NSAIDs can be very effective medications for arthralgia, arthritis or other rheumatic diseases, but their use in IBD is limited due to the risk of disease exacerbation and disease flares[32–38]. Even up to one-sixth of IBD patients in USA are chronically treated with opioids[39, 40]. Furthermore, pain in IBD was identified as the most common cause (48% of cases) for readmission 4 weeks after discharge from the hospital[41].

Here, we evaluated the well-characterized patient collective of the SIBDCS regarding pain, its impact of pain on daily life and social activities and the use of pain-specific medication. With 1’263 IBD patients included we present, to our best knowledge, the most extensive analysis of pain in IBD up to date.

## Methods

### Study Design

In the nationwide Swiss IBD Cohort Study (SIBDCS) patients with IBD from all regions of Switzerland have prospectively been included since 2006. In the SIBDCS at inclusion retrospective data is collected and afterwards patients are followed-up on a yearly basis. The cohort study is supported by the Swiss National Science Foundation and approved by the local ethical committees (IRB approval number: EK-1316, approved on 05.02.2007 by the Cantonal Ethics Committee of the Canton Zürich, Switzerland). As a participant of the SIBDCS the patients gave written informed consent for their clinical records to be used in this study. The cohort goals and methodology are described elsewhere[42].

2152 adult patients of the SIBDCS received a questionnaire regarding pain localization, impact of pain on daily life, how the surrounding responds to the patients’ pain and how activities of daily life are influenced. Furthermore, the questionnaire investigated the use of pain-specific medication. To develop the questionnaire, we used the validated German pain questionnaire[43]. Out of the 25 items in the German pain questionnaire 16 items were taken into our questionnaire (we used items 5, 6, 7, 8, 9, 10, 11, 12, 14, 15, 16, 18, 20, 21, 22, A1). Within these questions the patients were asked to mark their pain localizations in a body scheme and define the duration of the occurrence of pain (differentiating less than a month, 1 month– ½ year, ½ year– 1 year, 1–2 years, 2–5 years and >5 years). Furthermore, they had to define the pain attacks regarding duration, frequency, intensity and quality and the patients were questioned about medical and non-medical treatment of pain and the impact of pain on their duties of daily life and work. We used a German and a French version of the questionnaire. The questionnaires are included in the supporting information (S1 and S2 Files).

### Statistical Analysis

Clinical data were retrieved from the data center of the SIBDCS at the University of Lausanne. These data and additional data obtained from a review of the patients' files were entered into a database (Access 2000; Microsoft Switzerland Ltd Liab. Co., Wallisellen, Switzerland).

A descriptive statistical analysis was performed. Categorical variables were summarized as frequencies and percentages, whereas quantitative variables as median and range. Differences in categorical data distribution between groups were assessed using the Chi-squared test, or the Fisher’s exact test in case of insufficient sample size. The general wellbeing was analyzed by student’s t-test. For the analysis of the disease duration the Wilcoxon-Mann-Whitney ranksum test was used. A p-value < 0.05 was considered statistically significant. All statistical analyses were carried out using GraphPad Prism 5.04 for Windows (GraphPad Software Inc.).

## Results

### Patient’s characteristics

We received 1263 completed questionnaires (response rate 59%). 599 from 1263 of the patients were male (47%) and 664 were female (53%). The median age was 47 years. 679 patients had the diagnosis of CD (54%), 556 UC (44%), 28 indeterminate colitis (IC) (2%). EIM of IBD were present in 699 patients (55%). The median disease duration was 15 years (0–57 years) (Table 1). The mean disease duration of all IBD patients was 15 years (0–57 years). In a subgroup analysis the mean disease duration of CD patients was longer (16 years (0–57 years)) than in UC (14 years (0–49) which was statistically significant (p = 0.002). The last disease location can be seen in Table 1.

**Table 1:** Patient characteristics.

	Total IBD	Crohn’s Disease	Ulcerative colitis	
	N(%)	N(%)	N(%)	p-value
**Gender**				
Female	664 (53)	375 (55)	275 (49)	
Male	599 (47)	304 (45)	281 (51)	
**Diagnosis**	1263 (100)	679 (54)	556 (44)	
**Pain**				
Yes	894 (71)	484 (71)	388 (70)	p = 0.5726
No	369 (29)	195 (29)	168 (30)	
**EIM**				
Yes	699 (55)	421 (62)	266 (48)	
No	564 (45)	258 (38)	290 (52)	
**Disease duration**	**Years**			
Average	15			
Min-max	0–57			
**Last disease location**				
**Crohn’s disease (Montreal classification)**				
L1 only		186 (27)		
L1+L4		9 (1)		
L2 only		230 (34)		
L2+L4		9 (1)		
L3 only		198 (29)		
L3+L4		12 (2)		
L4 only		16 (2)		
Unknown/unclear		19 (3)		
**Ulcerative colitis**				
Left-sided colitis			231 (42)	
Pancolitis			189 (34)	
Proctitis			127 (23)	
Unknown/unclear			9 (2)	

### Prevalence of pain in IBD

A total of 1263 completed questionnaires was analyzed regarding pain. The vast majority of patients (894, 71%) reported having experienced pain in general during the course of the disease. Only 369 (29%) of the patients that sent back the questionnaire reported no pain (Table 1). There was no statistical difference when comparing CD and UC regarding the occurrence of pain (P = 0.5726) (Table 1). When comparing the prevalence of pain in patients with any extraintestinal manifestation (EIM) and without, slightly more patients with EIM (73%; 508 of 699 patients with EIM versus 68%; 386 of 564 patients without EIM) reported pain, but this did not reach statistical significance (p = 0.1058).

### Duration and evolution of pain in IBD

Pain was a longstanding problem for the majority of the patients with 52% (469 patients) of patients experiencing pain >5 years. Fifteen patients (2%) reported pain since <1 month, 57 patients (6%) suffered from pain since 1 month—½ year, 59 patients (7%) since ½ year– 1 year, 79 patients (9%) since 1–2 years and 215 (24%) since 2–5 years (Table 2). When comparing CD and UC 388 (70%) of the 556 UC patients reported pain in general. Of these the majority (191 patients, 49%) reported to suffer from pain more than 5 years. The 679 CD patients on the other hand also reported about pain in 71% (484 patients); of these a majority (265 patients, 55%) also suffered from pain >5 years. When comparing the duration of pain statistically more UC patients (47 patients; 12%) only suffered from pain in the last 1–2 years compared to CD (30 patients, 6%; p = 0.0026). For the other durations of pain there was no statistical difference (Table 2).

**Table 2:** Period suffering from pain.

	Total IBD	Crohn’s Disease	Ulcerative colitis	
Pain peroid	N(%)	N(%)	N(%)	p-value
<1 month	15 (2)	6 (1)	9 (3)	p = 0.2956
1 month—½ year	57 (6)	34 (7)	20 (5)	p = 0.4006
½ year-1 year	59 (7)	30 (6)	27 (7)	p = 0.6807
1–2 years	79 (9)	30 (6)	47 (12)	p = 0.0026
2–5 years	215 (24)	119 (25)	93 (24)	p = 0.8739
>5 years	469 (52)	265 (55)	191 (49)	p = 0.1166
No pain	369 (29.2)			

When characterizing the pain of all 894 IBD patients reporting of pain in general, 493 patients (55%) had pain attacks with no pain in between and 111 patients (12%) had pain attacks without being completely free of pain in between. 162 patients (18%) had a constant pain with slight fluctuations, 80 patients (9%) had constant pain with strong fluctuation. 48 patients (5%) did not specify (Table 3). When analysing the 484 CD patients who reported pain 268 patients (55%) reported about pain attacks with no pain in between, 67 (14%) had pain attacks without being completely free of pain in between. 91 (19%) had a constant pain with slight fluctuation, while 44 (9%) had a constant pain with strong fluctuation. When analysing the 556 UC patients 209 patients (54%) reported about pain attacks with no pain in between, 41 (11%) had pain attacks without being completely free of pain in between. 69 (18%) had a constant pain with slight fluctuation, while 34 (9%) had a constant pain with strong fluctuation. There was no statistically difference in the evolution of pain between CD und UC (Table 3).

**Table 3:** Pain characterization.

	Total IBD	Crohn’s Disease	Ulcerative colitis	
Pain character	N(%)	N(%)	N(%)	p-value
Constant pain w. slight fluctuation	162 (18)	91 (19)	69 (18)	p = 0.7253
Constant pain w. strong fluctuation	80 (9)	44 (9)	34 (9)	p = 0.9054
Pain attacks w. pain free intervals	493 (55)	268 (55)	209 (54)	p = 0.6815
Pain attacks w. constant pain	111 (12)	67 (14)	41 (11)	p = 0.1492
Not specified	48 (5)	14 (3)	34 (9)	p = 0.0002

### Frequency of pain in IBD

When characterizing the pain attacks of the 894 patients reporting pain in general, 173 patients (19%) had pain multiple times a day, 50 (6%) once daily, 137 (15%) multiple times per week, 38 (4%) once per week, 138 (15%) multiple times per month with only 73 patients (8%) reporting of pain once a month and 155 patients (17%) less than monthly. 130 patients (15%) did not specify (Table 4).

**Table 4:** Frequency of pain attacks.

	Total IBD	Crohn’s Disease	Ulcerative colitis	
Frequency of pain attacks	N(%)	N(%)	N(%)	p-value
Multiple daily	173 (19)	104 (21)	66 (17)	p = 0.1027
1x/day	50 (6)	26 (5)	21 (5)	p = 1.000
Multiple/week	137 (15)	86 (18)	49 (13)	p = 0.0385
1x/week	38 (4)	19 (4)	19 (5)	p = 0.5080
Multiple/month	138 (15)	66 (14)	64 (16)	p = 0.2518
1x/month	73 (8)	36 (7)	36 (9)	p = 0.3863
More seldom	155 (17)	82 (17)	69 (18)	p = 0.7872
Not specified	130 (15)	65 (13)	64 (16)	p = 0.2133

When comparing CD and UC patients in the group of CD patients presenting with pain 104 patients (21%) had pain multiple times a day, 26 (5%) once daily, 86 (18%) multiple times per week, 19 (4%) once per week, 66 (14%) multiple times per month, 36 (7%) once per month and 82 (17%) less than monthly. 65 (13%) did not specify. In the analysis of the UC patients 66 patients (17%) had pain multiple times a day, 21 (5%) once daily, 49 (13%) multiple times per week, 19 (5%) once per week, 64 (16%) multiple times per month, 36 (9%) once per month and 69 (18%) less than monthly. 64 patients (16%) did not specify. More CD patients (86 patients, 18%) reported of pain multiple times per week compared with UC (49 patients, 13%; p = 0.0385 (Table 4).

### Duration and intensity of pain episodes in IBD

The pain attacks most often had a duration of minutes (229 patients, 26%) to hours (244 patients, 27%), in 11% (102 patients) the pain duration was seconds and in 10% (93 patients) up to 3 days with only 73 patients (8%) reporting pain over more than 5 days. 153 patients (17%) did not specify the pain attacks (Table 5). In the subgroup analysis in the group of CD patients similar results were found with a pain duration of minutes (130 patients, 27%) to hours (124 patients, 26%), in 13% (63 patients) the pain duration was seconds and in 11% (55 patients) up to 3 days with 8% (38 patients) reporting pain over more than 5 days. 74 patients (15%) did not specify. In the group of UC patients there was a pain duration of minutes (92 patients, 24%) to hours (114 patients, 29%), in 10% (37 patients) the pain duration was seconds and in 9% (35 patients) up to 3 days with 8% (32 patients) reporting pain over more than 5 days. 78 patients (20%) did not specify. There was no statistically difference between CD und UC (Table 5).

**Table 5:** Duration of pain attacks.

	Total IBD	Crohn’s Disease	Ulcerative colitis	
Duration of pain attacks	N(%)	N(%)	N(%)	p-value
Seconds	102 (11)	63 (13)	37 (10)	p = 0.1341
Minutes	229 (26)	130 (27)	92 (24)	p = 0.3096
Hours	244 (27)	124 (26)	114 (29)	p = 0.2211
< 3 days	93 (10)	55 (11)	35 (9)	p = 0.2654
3 days	73 (8)	38 (8)	32 (8)	p = 0.9003
Not specified	153 (17)	74 (15)	78 (20)	p = 0.0723
Seconds	102 (11)	63 (13)	37 (10)	p = 0.1341
Minutes	229 (26)	130 (27)	92 (24)	p = 0.3096

The median pain intensity in the past 4 weeks was 2/10. 235 patients (26%) had no pain in the previous 4 weeks. The greatest pain intensity in the last 4 weeks was a median of 3/10.

### Pain localization

Most of the 894 patients who reported pain suffered from abdominal pain (532 patients, 59.5%), followed by back pain in 342 patients (38.3%), knee pain in 258 patients (28.9%) and hip pain in 231 patients (25.8%). 220 patients (24.6%) reported headaches, 132 patients neck pain (14.8%), 204 patients (22.8%) pain in the hand and finger joints, 90 patients (10.1%) reported pain in the elbows, 192 patients (21.5%) shoulder pain and 16.6% (148 patients) reported pain in the feet/ankles. 312 patients (34.9%) did not specify (Table 6).

**Table 6:** Pain localization.

	Total IBD	Crohn’s Disease	Ulcerative colitis	
Pain localization	N(%)	N(%)	N(%)	p-value
Head	220 (24.6)	123 (25)	92 (24)	p = 0.5807
Neck	132 (14.8)	75 (16)	56 (14)	p = 0.7033
Hand/finger	204 (22.8)	121 (25)	79 (20)	p = 0.1235
Elbow	90 (10.1)	52 (11)	36 (9)	p = 0.4991
Shoulder/arm	192 (21.5)	114 (24)	75 (19)	p = 0.1374
Back	342 (38.3)	195 (40)	139 (36)	p = 0.1836
Hip/thigh	231 (25.8)	132 (27)	95 (25)	p = 0.3931
Knee/ lower leg	258 (28.9)	143 (30)	113 (29)	p = 0.9404
Ankle/foot	148 (16.6)	84 (17)	61 (16)	p = 0.5831
Abdomen	532 (59.5)	293 (61)	224 (58)	p = 0.4062
Not specified	312 (34.9)	166 (34)	141 (29)	p = 0.5683

In the subgroup analysis there was no relevant difference in the pain localization in the CD and UC patients (Table 6).

### Treatment of pain

The majority of the 894 patients reporting of pain (600 patients, 67%), received pain medication. Only 116 patients (13%) had physiotherapy. 216 patients (24%) received no pain treatment.

When accessing the kind of medical pain treatment, the majority of 37% (333 patients) used Acetaminophen. Only 112 patients (13%) used NSAID and COX-2 inhibitors were used seldom (3%, 22 patients). Opioids or Metamizole were used in 16% (142 patients) while 239 patients (27%) did not specify (Table 7). In a subgroup analysis of the CD and UC patients reporting pain slightly more CD patients used Acetaminophen (39%, 189 patients) compared to UC (34%, 131 patients), but this was not statistically significant (p = 0.2918). For NSAIDs, COX-2 inhibitors, opioids and Metamizole there was no statistical difference when comparing CD and UC (Table 7).

**Table 7:** Treatment of pain.

	Total IBD	Crohn’s Disease	Ulcerative colitis	
Treatment of pain	N(%)	N(%)	N(%)	p-value
Acetaminophen	333 (37)	189 (39)	131 (34)	p = 0.1199
NSAID	112 (13)	63 (13)	45 (12)	p = 0.5370
Opioid/Metamizole	142 (16)	83 (17)	50 (13)	p = 0.0884
COX-2 inhibitor	22 (3)	16 (3)	6 (2)	p = 0.1283
Other	156 (17)			
Not specified	239 (27)	114 (24)	119 (31)	

### Impact of pain on quality of life

When assessing the impact on quality of life 528 patients (59%) of the patients had an impact on the duties of daily life, with a median of 3/10 (0: no impact, 10: very strong impact), 329 (37%) had no impact and 37 (4%) did not specify. 513 patients (57%) had an impact on their work with a median of 4/10 (0: no impact, 10: very strong impact), 344 (39%) had no impact on work, 37 (4%) did not specify. The general quality of life was significantly lower in patients suffering of pain compared to those without pain (38 vs. 77; (-100 very bad; 100 very good) p<0.0001).

## Discussion

Using data from 1,263 SIDBCS patients we showed prevalence of pain in IBD patients was high. With 71% of our patients reporting pain, pain is present in many more patients than generally assumed. This is consistent with data from Germany were a high prevalence of pain (87.9%) was found in a much smaller cohort of 334 patients[29]. When comparing CD and UC patients separately we could find no significant difference in the occurrence of pain. This is in line with the study by Schirbel et al. and also Heikenen et al. who evaluated presenting symptoms of IBD in a children cohort[29, 44].

For a significant number of patients with IBD chronic pain is a growing problem and has a large impact on quality of life. Due to its frequency and possible direct relation to a hypersensitivity state due to the inflammatory process, it has even been proposed to add chronic pain to the list of extraintestinal manifestations of IBD[39, 45].

Furthermore, we showed that pain is a longstanding problem for the majority of the patients affected. 52% of patients experiencing pain >5 years with only 2% reporting pain since less than 1 month. In a subgroup analysis comparing CD and UC, statistically more UC patients only suffered from pain in the last 1–2 years compared to CD (p = 0.0026). This reflects that pain in UC may be of shorter duration compared to CD. A possible explanation for this could be that CD patients, due to its higher prevalence of EIM, suffer more frequently of longer a duration of pain in comparison to UC [46]. In an evaluation of EIM in the SIBDCS by our group 43% of CD compared to 31% of UC patients had one to five EIMs[7]. Even though we could find that the disease duration in the UC patients was shorter than in the CD disease patients (14 years versus 16 years, p = 0.002), we don’t see this difference as relevant for the interpretation of the pain duration, especially regarding pain in the last 1–2 years.

The main pain localization was abdominal pain (59.5%), but a large proportion of patients also suffered from back pains, joint pains and headaches. Back pain was reported in 38.3% and 28.9% of patients suffered of knee pain. Furthermore 22.8% of IBD patients had pain in the hand and finger joints and 21.5% reported shoulder pain. This is in line with the literature showing that arthropathies are the most common extraintestinal manifestations in IBD[4, 6–16]. Of note the recognition of EIM is of great importance, since we could show that in one quarter of patients with IBD, EIMs appeared before the time of IBD diagnosis[47]. In a study by van der Have et al. IBD patients with back/joint pain reported a significantly lower quality of life and work productivity compared with IBD patients without back/joint pain[48]. In our cohort pain also had a strong impact on the health related quality of life (HRQOL). 59% of patients reported an impact on the HRQOL. Furthermore, HRQOL was significantly lower in patients suffering of pain as compared to those without pain (38 vs. 77; (-100 very bad; 100 very good) p<0.05.

When characterizing pain, the majority of patients reported pain attacks with no pain in between (55%). On the other hand, a substantial part of patients (40%) reported not being pain free between the pain attacks or even having a constant pain.

Notably, we show that pain is undertreated: one fourth of the patients reporting pain did not receive pain treatment. Given the high prevalence of joint and back pain and the know efficacy of physiotherapy it is surprising that only 13% of the patients received physiotherapy[49]. When accessing the different kinds of pain treatment, the majority received acetaminophen. However, a substantial part of patients was treated with NSAIDS despite that there is substantial evidence that there is a risk of exacerbation of IBD after treatment with NSAIDs. In a study by Takeuchi et al. nonselective NSAIDs were associated with a 17%-28% relapse rate within 9 days of ingestion[32–34].

Our study has strengths, but also limitations. A clear strength is the large cohort that we studied with 1263 completed questionnaires, making it, to our best knowledge, the largest study on this topic in IBD up to date.

The limitations of our study however, include that, due to the study design and the fact that we did not control unreturned questionnaires we can face a reporting bias. Patients who actually suffer from pain due to IBD might want to share their feelings, while patients that do not suffer would be more enticed to completely discard the questionnaire. This would result in overestimation of pain prevalence in our study. Against this limitation speaks, that compared to the German study by Schirbel et al. with 387 IBD patients, which had a response rate of 96.8%, our prevalence of pain was not higher[29].

In summary, using a nationwide patient cohort of IBD patients we have demonstrated that prevalence of pain in IBD patients is high and that it is present in many more patients than generally assumed. Further more pain has a substantial impact on the HRQOL. Thus, an increased awareness is mandatory to address this frequent complication in the course of IBD. Furthermore, it underlines the importance of pain management in IBD.

## Supporting Information

S1 File German SIBDCS Pain Questionnaire. Pain questionnaire sent to the patients of the Swiss IBD Cohort Study (SIBDCS) in German.
(DOCX)

S2 File French SIBDCS Pain Questionnaire. Pain questionnaire sent to the patients of the Swiss IBD Cohort Study (SIBDCS) in French.
(DOCX)
