# Supplementary material for: Pain in IBD Patients: Very Frequent and Frequently Insufficiently Taken into Account
Source: PLoS One. 2016 Jun 22;11(6):e0156666. doi: 10.1371/journal.pone.0156666 (PMC4917102; doi:10.1371/journal.pone.0156666)
Supplement: S1 File — Pain questionnaire sent to the patients of the Swiss IBD Cohort Study (SIBDCS) in German. (DOCX) [file pone.0156666.s001.docx]

**Schmerzfragebogen**

1. Patientennummer: _ _ _ _ _ _
2. Bitte zeichnen Sie im Körperschema ein, an welchen Körperstellen Ihre Schmerzen auftreten

Bitte beschreiben Sie Ihre Schmerzen mit eigenen Worten:

__________________________________________________________________________________________________________________________________________________________________________________________________________________________________________________________________________________________________________________________________________________________________________________________________________________________________________________________________

1. Wegen welcher Schmerzen kommen Sie hauptsächlich zur Behandlung?

_________________________________________________________________________________________________________________________________________________________________________________________________________________________________

1. a) **Seit wann** bestehen diese Schmerzen?

- weniger als 1 Monat
- 1 Monat bis ½ Jahr
- ½ Jahr bis 1 Jahr
- 1 bis 2 Jahre
- 2 bis 5 Jahre
- mehr als 5 Jahre

b) Können Sie ein **genaues Datum** angeben? (Tag I Monat I Jahr) I_ _I_ _I_ _ _ _I

1. a) Welche der Aussagen trifft auf Ihre Schmerzen in den letzten 4 Wochen am besten zu?

(Bitte nur eine Angabe machen!)

Zeit

Zeit

Zeit

Zeit

Schmerz

4) Schmerzattacken, auch dazwischen Schmerzen

3) Schmerzattacken, dazwischen schmerzfrei

2) Dauerschmerzen mit starken Schwankungen

1) Dauerschmerzen mit leichten Schwankungen

Wenn Sie an Schmerzattacken leiden (Bilder 3 und 4), beantworten Sie bitte noch zusätzlich folgende Fragen:

b) **Wie oft** treten diese Attacken durchschnittlich auf?

- mehrfach täglich
- einmal täglich
- mehrfach wöchentlich
- einmal wöchentlich
- mehrfach monatlich
- einmal monatlich
- seltener: ­­­­­­­­­­_____________________

c) **Wie lange** dauern diese Attacken durchschnittlich?

- Sekunden
- Minuten
- Stunden
- bis zu drei Tagen
- länger als drei Tage

1. Sind Ihre Schmerzen zu bestimmten Tageszeiten besonders stark?

- Ja
- Nein

Wenn ja:

- morgens
- mittags
- nachmittags
- abends
- nachts

1. Mit der folgenden Liste von Eigenschaftsworten können Sie genauer beschreiben, **wie Sie Ihre Schmerzen empfinden**. Denken Sie bei der Beantwortung an Ihre **typischen Schmerzen in der letzten Zeit**.

Bitte lassen Sie keine der Beschreibungen aus und machen Sie **für jedes Wort ein Kreuz**, inwieweit die Aussage für Sie zutrifft.

Sie haben bei jeder Aussage 4 Antwortmöglichkeiten:

3 = trifft genau zu 2 = trifft weitgehend zu 1 = trifft ein wenig zu 0 = trifft nicht zu

Ich empfinde die Schmerzen als…

I

|  | Trifft genau zu | Trifft weitgehend zu | Trifft ein wenig zu | Trifft nicht zu |
| --- | --- | --- | --- | --- |
|  | 3 | 2 | 1 | 0 |
| …dumpf | O | O | O | O |
| …drückend | O | O | O | O |
| …pochend | O | O | O | O |
| …klopfend | O | O | O | O |
| …stechend | O | O | O | O |
| …heiss | O | O | O | O |
| …brennend | O | O | O | O |
| …elend | O | O | O | O |
| …schauderhaft | O | O | O | O |
| …scheusslich | O | O | O | O |
| …furchtbar | O | O | O | O |

SBL © Korb 2006

1. Geben Sie im Folgenden die **Stärke Ihrer Schmerzen** an. Kreuzen Sie **auf den unten aufgeführten Linien** an, wie stark Sie Ihre Schmerzen empfinden (unter Ihrer üblichen Medikation). Die Zahlen können Ihnen bei der Einteilung helfen: Ein Wert von 0 bedeutet, Sie haben keine Schmerzen, ein Wert von 10 bedeutet, Sie leiden unter Schmerzen, wie sie für Sie nicht stärker vorstellbar sind. Die Zahlen dazwischen geben Abstufungen der Schmerzstärke an.
2. Geben Sie bitte zunächst Ihre **momentane Schmerzstärke** an:


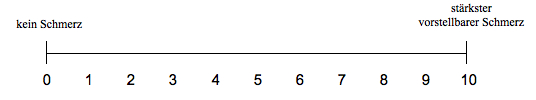


1. Geben Sie jetzt bitte Ihre **durchschnittliche Schmerzstärke** während der letzten 4 Wochen an:


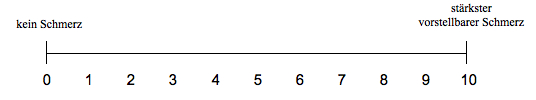


1. Geben Sie jetzt bitte Ihre **größte Schmerzstärke** während der letzten 4 Wochen an:


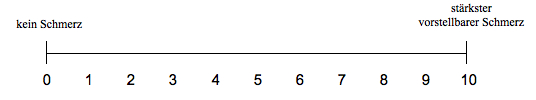


1. Geben Sie jetzt an, welche **Schmerzstärke** für Sie bei erfolgreicher Behandlung **erträglich** wäre:


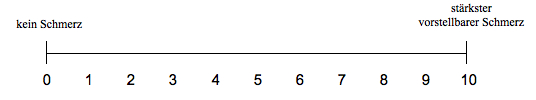


1. In den folgenden Fragen geht es um Ihre Schmerzen während der **letzten 3 Monate**. Für diesen Zeitraum möchten wir Genaueres über die **Auswirkungen der Schmerzen** erfahren.
2. **An wie vielen Tagen** konnten Sie in den letzten 3 Monaten aufgrund von Schmerzen nicht Ihren üblichen Aktivitäten nachgehen (z.B. Beruf, Schule, Haushalt)?

an etwa I_I_I Tagen

1. In welchem Maße haben die Schmerzen in den letzten 3 Monaten Ihren **Alltag** (Ankleiden, Waschen, Essen, Einkaufen etc.) beeinträchtigt?


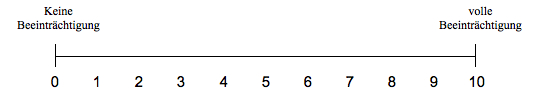


1. In welchem Maße haben die Schmerzen in den letzten 3 Monaten Ihre **Freizeitaktivitäten** oder Unternehmungen im **Familien- oder Freundeskreis** beeinträchtigt?


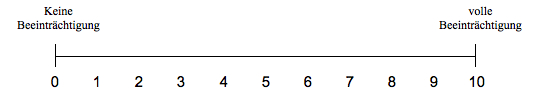


1. In welchem Maße haben die Schmerzen in den letzten 3 Monaten Ihre **Arbeitsfähigkeit** (einschließlich Hausarbeit) beeinträchtigt?


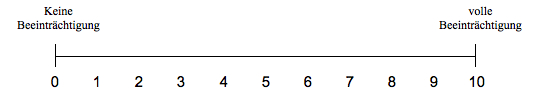


1. Was machen Sie selbst, um Ihre **Schmerzen günstig zu beeinflussen**? Bitte machen Sie genaue Angaben, z.B. spazieren gehen, schlafen, Ablenkung, ...

_________________________________________________________________________________________________________________________________________________________________________________________________________________________________

- Ich kann meine Schmerzen nicht beeinflussen

1. **Was löst** Ihrer Erfahrung nach **die Schmerzen aus oder verschlimmert** sie?

_________________________________________________________________________________________________________________________________________________________________________________________________________________________________

- Ich weiss es nicht

1. Bitte schätzen Sie Ihr derzeitiges allgemeines Wohlbefinden ein. Geben Sie bitte an, wie Sie sich in den letzten 14 Tagen meistens gefühlt haben. Kreuzen Sie dazu auf der 6-stufigen Skala jeweils die Zahl an, die am ehesten auf Sie zutrifft: 0 = trifft gar nicht zu, 5 = trifft vollkommen zu. Bearbeiten Sie bitte alle Aussagen.

FW7 © Herda, Scharfenstein u. Basler 1998

| Trotz der Schmerzen würde ich sagen: | trifft gar nicht zu |  |  |  |  | trifft vollkommen zu |
| --- | --- | --- | --- | --- | --- | --- |
|  | **0** | **1** | **2** | **3** | **4** | **5** |
| 1. Ich habe meine alltäglichen Anforderungen im Griff gehabt. | O | O | O | O | O | O |
| 2. Ich bin innerlich erfüllt gewesen. | O | O | O | O | O | O |
| 3. Ich habe mich behaglich gefühlt. | O | O | O | O | O | O |
| 4. Ich habe mein Leben genießen können. | O | O | O | O | O | O |
| 5. Ich bin mit meiner Arbeitsleistung zufrieden gewesen. | O | O | O | O | O | O |
| 6. Ich war mit meinem körperlichen Zustand einverstanden. | O | O | O | O | O | O |
| 7. Ich habe mich richtig freuen können. | O | O | O | O | O | O |

1. Ich denke des Öfteren daran, mir das Leben zu nehmen
   - Ja
   - Nein
2. **Wie** wurden Ihre Schmerzen **bisher behandelt**?

Kreuzen Sie bitte an, welche der unten aufgeführten Behandlungsmaßnahmen Sie erhalten haben.

- bisher **keine** Schmerzbehandlung
- Medikamente
- Infusionen
- Einspritzungen in das Schmerzgebiet, Nervenblockaden
- Einspritzungen am Rückenmark (z.B. epidural)
- Rückenmarksnahe Sonden- (SCS) oder Pumpensysteme
- Krankengymnastik
- Massagen, Bäder, Kälte-/Wärmetherapie
- Elektrische Nervenstimulation (TENS)
- Akupunktur
- Chiropraktik
- Psychotherapie
- Entspannungsverfahren, Hypnose, Biofeedback
- Medikamenten-Entzug
- Kur-/Reha-Behandlung
- Anderes: _____________________________________

1. **Schmerz-Medikamenten-Einnahme**. Bitte tragen Sie in die nachfolgende Tabelle alle Schmerz-Medikamente ein, die Sie zur Zeit verwenden

| Bitte Ihre Medikamente hier eintragen: | Ich nehme das Medikament | | | |  |
| --- | --- | --- | --- | --- | --- |
|  | mehrfach täglich | einmal täglich | mehrfach wöchentlich | Mehrfach im Monat | seltener |
|  | O | O | O | O | O |
|  | O | O | O | O | O |
|  | O | O | O | O | O |
|  | O | O | O | O | O |
|  | O | O | O | O | O |
|  | O | O | O | O | O |
|  | O | O | O | O | O |
|  | O | O | O | O | O |
|  | O | O | O | O | O |

1. Wurden Sie schon einmal **operiert**?

- Ja, I_I_I mal (wie oft)
- Nein

Wichtig sind für die Beantwortung dieser Frage auch alle „kleineren operativen Eingriffe“, die oft in örtlicher Betäubung durchgeführt werden, z.B. Nasenoperationen, Gelenk- und Bauchspiegelungen. Bitte markieren Sie, welche Operation **wegen Ihrer Schmerzen** durchgeführt wurde.

Wegen Schmerz?

1. ________________________________ Datum: I_I_I I_I_I I_I_I_I_I O
2. ________________________________ Datum: I_I_I I_I_I I_I_I_I_I O
3. ________________________________ Datum: I_I_I I_I_I I_I_I_I_I O
4. ________________________________ Datum: I_I_I I_I_I I_I_I_I_I O
5. ________________________________ Datum: I_I_I I_I_I I_I_I_I_I O
6. Die Folgende Frage bezieht sich auf die **letzten 14 Tage**:

Wie war Ihr **allgemeines Wohlbefinden**? Ordnen Sie Ihrem Befinden eine Position auf der Linie zu, wobei „-100“ einem sehr schlechten Befinden und „+100“ einem sehr guten Befinden entspricht. Machen Sie eine Markierung an der Stelle, die Ihrem Befinden entspricht.

sehr schlecht sehr gut

-100 0 +100
