# Supplementary material for: Pain in IBD Patients: Very Frequent and Frequently Insufficiently Taken into Account
Source: PLoS One. 2016 Jun 22;11(6):e0156666. doi: 10.1371/journal.pone.0156666 (PMC4917102; doi:10.1371/journal.pone.0156666)
Supplement: S2 File — Pain questionnaire sent to the patients of the Swiss IBD Cohort Study (SIBDCS) in French. (DOCX) [file pone.0156666.s002.docx]

**Formulaire d’évaluation de la douleur**

1. Numéro de patient: _ _ _ _ _ _
2. Représentez sur ce schéma corporel les diverses localisations de vos douleurs

Décrivez ces douleurs avec vos propres mots:

__________________________________________________________________________________________________________________________________________________________________________________________________________________________________________________________________________________________________________________________________________________________________________________________________________________________________________________________________

1. Citez les douleurs principales vous amenant à consulter

_________________________________________________________________________________________________________________________________________________________________________________________________________________________________

1. a) **Depuis quand** avez-vous ces douleurs ?
   - - moins d’un mois
     - 1 à 6 mois
     - 6 mois à 1 an
     - 1 à 2 ans
     - 2 à 5 ans
     - plus de 5 ans

b) pouvez-vous donner une **date exacte d’apparition** de la douleur?

(jour/mois/année) __/__/____

1. a) Parmi les descriptions ci-dessous, choisissez celle qui définit le mieux vos douleurs au cours des 4 dernières semaines (une seule réponse) :

le temps

le temps

le temps

le temps

douleur

1) douleurs continues avec de légères variations

4) douleurs aigües sur un fond de douleurs constantes

3) douleurs aigües suivies d’intervalles sans douleurs

2) douleurs continues avec de fortes variations

Si vous souffrez de douleurs aigües (schéma 3 et 4), répondez aux questions suivantes :

b) **à quelle fréquence** en moyenne surviennent ces douleurs aigües?

- plusieurs fois par jour
- 1x par jour
- plusieurs fois par semaine
- 1x par semaine
- plusieurs fois par mois
- 1x par mois
- rarement :_________

c) **combien de temps** durent ces douleurs aigües?

- quelques secondes
- quelques minutes
- quelques heures
- jusqu’à 3 jours
- plus de 3 jours

1. Vos douleurs sont-elles particulièrement fortes à un moment donné de la journée ?

- Oui
- Non

Si oui, quand ?

- le matin
- vers midi
- l’après-midi
- en soirée
- la nuit

1. Au moyen des adjectifs qualificatifs listés ci-dessous, décrivez exactement **comment vous ressentez vos douleurs**. En répondant, pensez aux **douleurs typiques éprouvées ces derniers temps.**

Ne laissez pas de cases vides, **faites une croix pour chaque adjectif** dans la mesure où celui-ci correspond à la description de vos douleurs.

Vous avez 4 possibilités de réponse pour chaque énoncé :

Mes douleurs ont un caractère…

I

|  | Correspond  exactement | Correspond en grande partie | Correspond un peu | Ne correspond pas |
| --- | --- | --- | --- | --- |
|  | 3 | 2 | 1 | 0 |
| ... sourd | O | O | O | O |
| …oppressant | O | O | O | O |
| …pulsatile | O | O | O | O |
| …battant | O | O | O | O |
| …piquant | O | O | O | O |
| …chaud | O | O | O | O |
| …brûlant | O | O | O | O |
| …misérable | O | O | O | O |
| …affreux | O | O | O | O |
| …abominable | O | O | O | O |
| …terrible | O | O | O | O |

SBL © Korb 2006

1. Indiquez sur l’échelle ci-dessous **l’intensité de vos douleurs** (sous votre médication habituelle), à l’aide de la gradation proposée :

0= pas de douleurs, 10= douleurs les plus fortes pouvant être imaginées.

Les chiffres entre ces 2 extrêmes indiquent une gradation de l’intensité de vos douleurs.

a) Décrivez l’intensité de douleur que vous ressentez **actuellement**


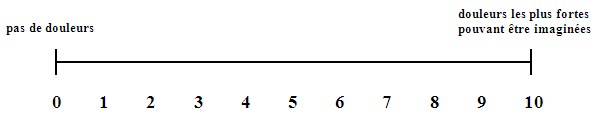


b) Décrivez l’intensité de douleur **moyenne au cours des 4 dernières semaines**


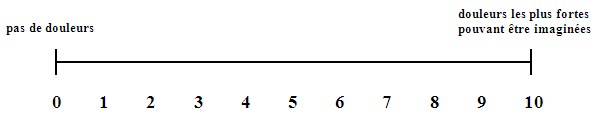


c) Décrivez l’intensité de douleur **maximale** ressentie au cours des 4 dernières semaines


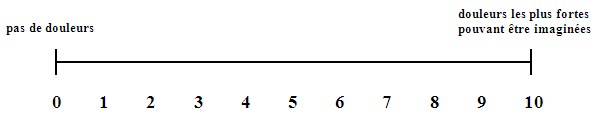


d) Décrivez **l’intensité de douleur qui serait supportable** dans le cas d’un traitement considéré comme efficace


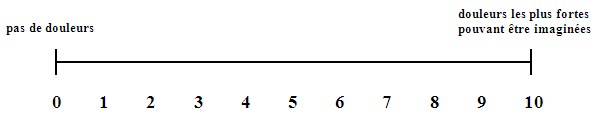


1. Les questions suivantes concernent vos douleurs **au cours des 3 derniers mois**. Nous souhaitons évaluer les **conséquences de ces douleurs sur votre vie quotidienne** durant cette période
2. Au cours des 3 derniers mois, **pendant combien de jours** avez-vous dû renoncer à vos activités habituelles (p.ex. profession, école, ménage) ?

Environ ___ jours

1. Au cours des 3 derniers mois, dans quelle mesure **votre vie quotidienne** (s’habiller, faire sa toilette, préparer les repas, faire des achats) a-t-elle été altérée par les douleurs ?

0= pas d’altération, 10= incapacité/altération totale


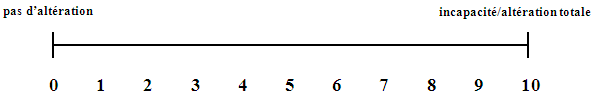


1. Au cours des 3 derniers mois, dans quelle mesure vos **activité de loisirs** ou **activités en famille/avec des amis** ont-elles été altérées ?


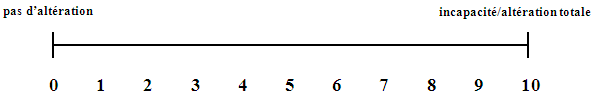


1. Au cours des 3 derniers mois, dans quelle mesure votre **capacité de travail** (y compris la tenue du ménage) a-t-elle été altérée ?


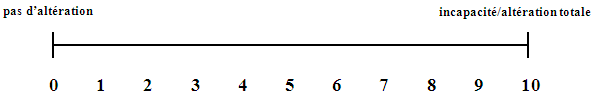


1. Que faites-vous **vous-même spontanément afin de réduire vos douleurs** ?

Veuillez donner des exemples précis : p.ex. se promener, dormir, se distraire, …

_________________________________________________________________________________________________________________________________________________________________________________________________________________________________________________________

- Je ne peux pas influencer mes douleurs

1. Selon votre propre expérience, **par quoi les douleurs sont-elles déclenchées ou aggravées**? ____________________________________________________________________________________________________________________________________________________________________________________________________________________________________________________________

- Je ne sais pas

1. Evaluez votre état de bien-être. Indiquez comment vous vous êtes sentis en général au cours des 2 dernières semaines. Indiquez au moyen d’une croix sur le tableau ci-dessous le chiffre qui vous correspond le mieux : 0= ne correspond pas du tout, 5= correspond totalement. Répondez à tous les énoncés.

FW7 © Herda, Scharfenstein u. Basler 1998

| Malgré les douleurs, je considère: | Ne correspond pas du tout |  |  |  |  | Correspond totalement |
| --- | --- | --- | --- | --- | --- | --- |
|  | **0** | **1** | **2** | **3** | **4** | **5** |
| 1. avoir accompli mes tâches quotidiennes | O | O | O | O | O | O |
| 2. être épanoui intérieurement | O | O | O | O | O | O |
| 3. m’être senti à l‘aise | O | O | O | O | O | O |
| 4. avoir pu profiter de la vie | O | O | O | O | O | O |
| 5. avoir été satisfait de ma performance au travail | O | O | O | O | O | O |
| 6. avoir été satisfait de mon état corporel | O | O | O | O | O | O |
| 7. avoir pu me réjouir véritablement | O | O | O | O | O | O |

1. Je pense souvent au suicide

- oui
- non

1. **Comment** vos douleurs ont-elles **été traitées jusqu’à présent** ?

Parmi les traitements mentionnés ci-dessous, indiquez au moyen d’une croix ceux que vous avez déjà reçu.

- **Pas de thérapie** anti-douleur jusqu’à présent
- Médicaments
- Perfusion (administration intraveineuse)
- Injections à l’endroit douloureux, bloc nerveux
- Injections au niveau de la moelle épinière (p.ex. injection épidurale)
- Stimulation de la moelle épinière (SCS) ou pompe anti-douleur (PCA)
- Physiothérapie
- Massages, bains, thérapie chaud/froid
- Neurostimulation électrique transcutanée (TENS)
- Acupuncture
- Chiropracticien/ostéopathie
- Psychothérapie
- Techniques de relaxation, hypnose, Biofeedback
- Sevrage médicamenteux
- Cure/séjour de réhabilitation
- Autres :____________

1. **Prise de médicaments anti-douleurs** : inscrivez dans la tabelle ci-dessous tous les médicaments anti-douleurs que vous prenez actuellement.

| Nom du médicament: | Je prends ce médicament: | | | |  |
| --- | --- | --- | --- | --- | --- |
|  | Plusieurs fois par jour | 1x par jour | Plusieurs fois par semaine | Plusieurs fois par mois | Rarement |
|  | O | O | O | O | O |
|  | O | O | O | O | O |
|  | O | O | O | O | O |
|  | O | O | O | O | O |
|  | O | O | O | O | O |
|  | O | O | O | O | O |
|  | O | O | O | O | O |
|  | O | O | O | O | O |
|  | O | O | O | O | O |

1. Avez-vous déjà été **opéré(e)** ?

- Oui : ___fois
- Non

Il est important de mentionner dans la réponse à cette question également toutes les « petites opérations », souvent réalisées en anesthésie locale (p.ex. opération du nez, opération des articulations, gastroscopie, …).

Précisez quelle(s) opération(s) a/ont été faite(s) **dans le but de soulager vos douleurs**.

Afin de soulager vos douleurs ?

1. ________________________________ Date: I_I_I I_I_I I_I_I_I_I O
2. ________________________________ Date: I_I_I I_I_I I_I_I_I_I O
3. ________________________________ Date: I_I_I I_I_I I_I_I_I_I O
4. ________________________________ Date: I_I_I I_I_I I_I_I_I_I O
5. ________________________________ Date: I_I_I I_I_I I_I_I_I_I O
6. Cette question porte sur les **2 dernières semaines** :

Comment évaluez-vous votre **bien-être général** durant les 2 dernières semaines ? Indiquez au moyen d’une croix sur l’échelle ci-dessous l’état dans lequel vous estimez vous trouver.

« -100 » : très mauvais état de santé

« +100 » : excellent état de santé

Très mauvais état de santé Excellent état de santé

-100 0 +100
